# Charleston Medical and Surgical Journal

**Published:** 1858-09

**Authors:** 


Charleston Medical and Surgical Journal.—A very decided improvement has taken place in the scientific and literary character of this Journal, as well as in its tone, since it has fallen into the hands of its present able Editor. The July number contains a number of original articles and reviews of marked ability. The work is evidently destined to take a high rank among the medical journals of the country.

